# Defending LGBTQIA+ Aging Research: U.S. Federal Policies’ Impacts on Studies of Older Sexual and Gender Minorities

**DOI:** 10.1093/geroni/igaf122.439

**Published:** 2025-12-31

**Authors:** Jace Flatt, Joel Anderson, Whitney Wharton

**Affiliations:** University of Nevada Las Vegas, Nevada, United States; University of Tennessee, Knoxville, Tennessee, United States; Emory University, Atlanta, Georgia, United States

## Abstract

Funding supporting research about sexual and gender minority aging and Alzheimer’s disease and related dementias is currently being terminated by the U.S. federal government. During the past year, research grants that included older transgender people, as well as lesbian, gay, bisexual, queer, intersex, and additional identities (LGBTQIA+), have been terminated, funding decisions rescinded, and grants pulled from scientific review. Recent termination letters from the federal government have claimed that “research programs based on gender identity are often unscientific, have little identifiable return on investment, and do nothing to enhance the health of many Americans. Many such studies ignore, rather than seriously examine, biological realities.” This presentation will highlight the impact of anti-LGBTQIA+ policies on the current state of science and explore potential action steps and strategies that researchers committed to supporting and conducting research with sexual and gender minority aging populations should consider. We will highlight our efforts to advocate for this research, including talking to the media, writing to policymakers, generating visibility on social media with colleagues and peers, speaking with lawyers and legal scholars, and connecting with community members. This includes a discussion of several case studies involving research with LGBTQIA+ older adults that has been abruptly terminated by the federal government. We will also discuss ways to support our colleagues, junior faculty and early career scholars, conduct emancipatory research, and stand up against current policies to abrogate diversity, equity, inclusion, and accessibility, as well as anti-transgender ideologies in research and academia.

